# Sodium and Hematocrit Levels’ Correlation and Clinical Impacts in Jordanian Hemodialysis Patients

**DOI:** 10.7759/cureus.71208

**Published:** 2024-10-10

**Authors:** Mahmoud H Al Hindawi, Muhannad M Asi, Mohammad A Obeidat, Mousa Atmeh, Thaer J Al Kasasbeh

**Affiliations:** 1 Nephrology Department, Jordanian Royal Medical Services, Amman, JOR; 2 Internal Medicine Department, Jordanian Royal Medical Services, Amman, JOR; 3 Clinical Oncology Department, Jordanian Royal Medical Services, Amman, JOR

**Keywords:** health care outcomes, hematocrit to hemoglobin ratio, hyponatraemia, intermittent hemodialysis (ihd), jordanian, optimal threshold

## Abstract

Aim: Given cofounders, this retrospective study investigates the correlation between hyponatremia status and hematocrit (Hct) changes, as well as the clinical utility of these two prognosticators in relation to overall health status.

Methods: The study was retrospectively conducted on adult hemodialysis (HD) patients at the King Hussein Medical Center in Amman, Jordan, from 2015 to 2022. It looked at how sodium (Na) levels, the hematocrit-to-hemoglobin ratio (HHR), and outcomes of interest were related. The study examined acute myocardial infarction, stroke, refractory hypertension, dialysis graft thrombosis, and all-cause mortality as the composite outcomes of interest (cOI). We conducted a series of receiver operating characteristics, binary logistic regression (BLgR), and sensitivity analyses between each tested prognosticator and the cOI. We constructed and illustrated a multiple logistic regression (MLgR) model to investigate the adjusted association of each prognosticator against the tested composite outcome probability.

Results: The majority of HD patients were hemolyzed for a period of four to seven years. The constructed binary regression modeling for each prognosticator was [e (109.36 - 0.849 × Na)/[1 + e (109.36 - 0.849 × Na)] and [e (16.033 - 6.388 × HHR)/[1 + e (16.033 - 6.388 ×HHR)], with a 35.57% probability of cOI at the optimal Na of 129.51 mEq/l and a 42.8% at optimal HHR of 2.555:1. When the length of hemodialysis (LOD) was introduced into the MLgR modeling all the investigated independent variables were significant except the HHR (p-value = 0.908).

Conclusion: A higher LOD, lower Na, and lower HHR all support a positive cOI probability status. Regular inspection of Na and HHR and ensuring their closet to their optimal thresholds are mandatory to mitigate the risk of higher cOI probability.

## Introduction

Dialysis is a complicated process that removes soluble blood waste with plasma water-derived dialysate. Patients and technology customize the dialysate for extracorporeal renal replacement therapy (RRT). Dialysis uses small filtering surfaces, impermeable cellulosic membranes, and open reservoirs [1-2].

In the 1960s and 1970s, diffusion and osmosis removed interdialytic salt and water accumulations instead of hydrostatic transmembrane pressure, or "ultrafiltration." Dialysis systems have evolved to improve RRT safety and efficacy. If the sodium (Na) level in dialysis is less than 126 mEq/L, it is likely that 70%-90% of patients will experience intradialytic hypotension and gain weight between dialysis sessions [3-4].

Beyond the 1970s, the efficiency of dialyzers increased, leading to the introduction of hydrostatic transmembrane pressure-driven ultrafiltration. The potentially fatal "disequilibrium" syndrome caused lethargy, fatigue, nausea, headaches, and muscle cramps. Limiting dialysis output or using osmotic agents can treat brain edema, the most serious complication caused by a sudden drop in serum urea or osmolality. Durable dialyzers, blood pumps, and negative-pressure systems improved hemolysis [5-6].

In 1980, mechanical hydrostatic fluid removal exceeded 1 L/hour. This managed uremia, salt, and water during a short and effective hemolysis session. This reduces treatment times and eases patient rehabilitation. By increasing plasma volume and tissue perfusion, dialysis reduces pain [7].

Dialysate Na increased from 1980 to 1995, lowering the risk of blood urea nitrogen (BUN) and cerebral edema. High-Na dialysate reduces dialysis disequilibrium by removing intracellular, interstitial, and plasma water. However, Na-rich bicarbonate dialysate offers a more comfortable rate and hemodynamic stability. It was believed that abrupt changes in plasma osmolarity caused dialysis disequilibrium and hemodynamic instability [8]. Dialysate tonicity was helpful. High-Na dialysate prevents tissue hypoperfusions, reducing dialysis symptoms like nausea, vomiting, headache, chest pain, hypotension, and cramps. Weight gain and high blood pressure occur before and during high-efficiency dialysis with high-Na dialysate. Short, high-efficiency dialysis may be uncomfortable due to stress [9].

Dialysis helps hemodialysis (HD) patients manage the mineral-bone disease, uremic toxins, and anemia, which affect prognosis. Most research focuses on stable HD patients who can do daily tasks. Researchers have looked at things that can affect a person's prognosis, like diabetes, low-Na levels, hemodilution, acute myocardial infarction, stroke, interdialytic refractory hypertension, intradialytic hypotension, and graft thrombosis in people who are on stable hemodilution [10-11].

Indeed, mortality rates are higher in hypovolemic advanced hemolysis patients who have a longer HD length. The unknown causes of hyponatremia-related mortality complicate treatment with personalized dialysate Na prescriptions. To reduce the associated risks, identify and address the causes of hyponatremia. However, keeping the Na concentration close to the ideal level, even when the blood Na level is low, might also lower the risk of complications related to low Na [12-13].

Noninvasive blood flow measurements through dialyzer lines estimate blood volume (BV) changes. The majority of methods measure hematocrit (Hct), whole blood hemoglobin (Hgb), and total blood protein. This method assumes that dialysis maintains the number of red blood cells (RBCs) in the circulatory system, as well as the whole-blood-to-central Hct ratio. It is necessary to monitor Hct, Hgb, or the Hct-to-Hgb ratio (HHR) for BV estimation in HD patients who require regular maintenance. However, as water moves from RBCs to plasma, the arterial bloodline Hct may not accurately reflect BV drops [14-15].

In this study, we initially aimed to investigate the ideal thresholds for Na and HHR, which were associated with the positive outcomes of our Jordanian HD patients, while also examining their prognostic effectiveness and sensitivity. We then created a multiple logistic regression model that combined the adjusted relationships between these tested predictors and the probability of at least one of the planned bad composite outcomes of interest (cOI).

## Materials and methods

The King Hussein Medical Center (KHMC) at Royal Medical Services (RMS) in Amman, Jordan, enrolled 926 patients in this observational single-center retrospective study from 2015 to 2022. Our Institutional Review Board (IRB) requested ethical approval under approval number 17_10/2023 to review medical records from the Hakeem system for 438 HD patients who continued HD after 2018 until the end of data collection (December 2022), including department paper records and sheets for 488 HD patients who started HD sessions prior to or in 2015 until 2018.

For a minimum of six months, all patients received four-hour HD sessions twice or three times per week. Individuals who had received peritoneal dialysis or a kidney transplant were not eligible. Adults over the age of 18 were considered. People with chronic kidney disease (CKD), proteinuria, hematuria, or structural renal disease were included in our study. They could have an estimated glomerular filtration rate of 60 mL/min/1.73 m^2^ or less. The study included all HD patients whose medical records contained at least 80% of the required information.

We collected patient demographic data, including age, gender, HD duration, comorbidity burden, interdialytic weight gain, and the existence of any cOI under investigation. From blood samples taken from the arteriovenous shunt, we carefully got up to two digits of the cumulative mean predialysis biochemical parameters for specific Na, Hct, and Hgb levels. We manually calculated the Hct and Hgb-based quotient (HHR). This study assessed the cOI positivity by assessing the occurrence of at least one of the following poor outcomes: acute myocardial infarction, stroke, resistant hypertension, dialysis graft thrombosis, or all-cause mortality.

First, we performed a binary logistic regression (BLgR) analysis to examine the regressional associations between the patients' Na or HHR values and their assessed cOI. This binary analysis sought to ascertain the strength of the correlations, the percentage of the dependent variable's variability that the independent variables explained, and the precision of the dependent variable's prediction. To find out how Na and HHR, the predictors we were looking into, affected the chance of cOI events, we then did receiver operating characteristic (ROC) curve analyses and sensitivity analyses. These events could either involve no occurrence of any aforementioned cOI (represented as negative or 0), or they could involve at least one occurrence of cOI (represented as positive or 1).

Subsequently, we pursued serial multiple logistic regression (MLgR) analyses that adopted both of this study’s primary investigated prognosticators in two opposite scenarios. In the initial scenario (Model I), we viewed HHR as a parametric variable and Na as a dichotomized variable. We classified Na as either lower than its optimal point of 129.51 mEq/l, which we signed as 0, or higher than 129.51 mEq/l, which we signed as 1. In the second scenario (Model II), we take a different approach, treating Na as a parametric variable. Meanwhile, we dichotomize the HHR into two categories: lower than the explored optimal point (2.555), signifying 0 or higher than 2.555, indicating 1. In the third scenario (Model III), we considered both Na and HHR as binary variables, dichotomized as previously mentioned in Model I and Model II, respectively. However, the length of HD was considered as a third independent variable in the multiple logistical regression analyses we conducted. By creating Model III, our goal was to show how important both of our main tested predictors were for a patient's chance of cOI, while also taking into account how important the patient's length of HD was. This study used MS Excel (Microsoft Corporation, Redmond, Washington, United States) for data collection and IBM SPSS Statistics for Windows, Version 25 (Released 2017; IBM Corp., Armonk, New York, United States) for analysis. This study adopted a significance level of 0.05.

## Results

The KHMC at RMS in Amman, Jordan conducted an observational study using a retrospective approach, with a total of 1306 patients participating in the hemodialysis unit (HDU). The study's course lasted from 2015 until 2022. This study excluded a total of 380 patients due to factors such as missing data (more than 20%) and incorrect national or military identification numbers, particularly for those who started or continued HD between 2015 and 2018 and later died or underwent kidney transplantation. This occurred because the provided information was either incomplete or inaccurate.

We reviewed the medical records from the Hakeem system for 47.3% of the 438 HD patients who began or continued HD after 2018 until the end of the data collection period in December 2022. We conducted this review in compliance with the Medical Review Board's (IRB) ethical approval number 17_10/2023. In addition, we examined 52.7% of the department's paper records and sheets, which included 488 HD patients, for patients who had begun or continued their HD sessions between the years 2015 and 2018.

There were approximately 70.7% of HD patients, or 655 individuals, who did not experience any of the following during their length of hemodialysis (LOD) years: acute myocardial infarction, stroke, resistant hypertension, dialysis graft thrombosis, or all-cause mortality (with a significantly improved cOI). Conversely, of the 271 HD patients, 29.3% (n = 271) showed at least one of the previously mentioned COIs, a significantly lower percentage. For the purpose of this investigation, there were a total of 646 males with HD and 30.2% (280 females with HD) males who participated.

Based on the Na levels and the HHR that were studied, BLgR-based models were used to estimate the percent chance of having a positive cOI over a negative cOI. The models came up with [e (109.36 - 0.849 Na)/[1 + e (109.36 - 0.849 Na)] and [e (16.033 - 6.388 HHR)/[1 + e (16.033 - 6.388 HHR)]. Furthermore, the study achieved correct classification rates of 82.2% (χ2(8) = 42.344; p-value = 0.000) and 70.8% (ꭓ2(8) = 63.329; p-value = 0.000) for the cases, respectively.

**Figure 1:**
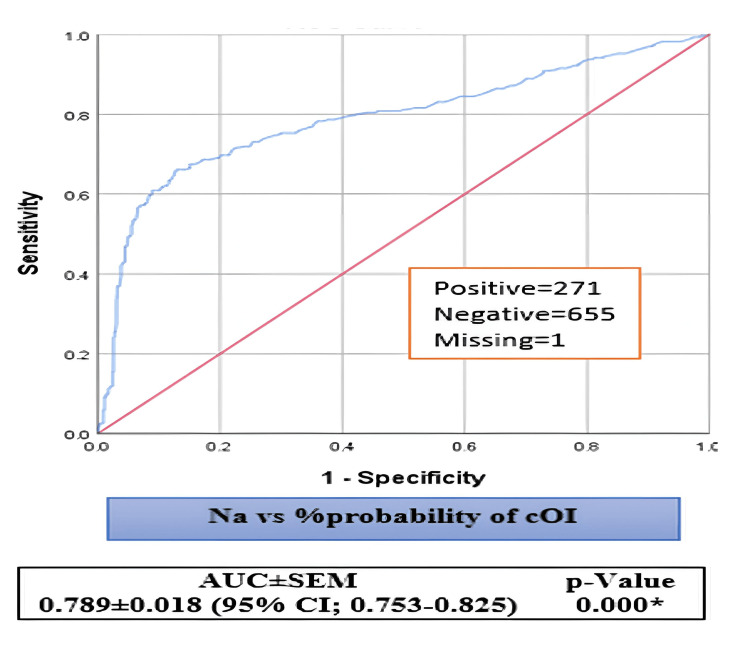
The receiver operating characteristic (ROC) test for Na levels vs. probability of cOI positivity Na: Sodium; cOI: composite outcomes of interest; ROC: receiver operating characteristic; SEM: standard error of mean; CI: confidence interval; *: statistically significant; AUROCs: area under the ROC curves The ROC test examined the AUROCs for Na levels against the cOI. Higher %Prob of cOI values indicate stronger positive evidence for our tested prognosticators. The lower values of the independent variables suggest a more negative state (higher %Prob of naïve cOI)

**Figure 2:**
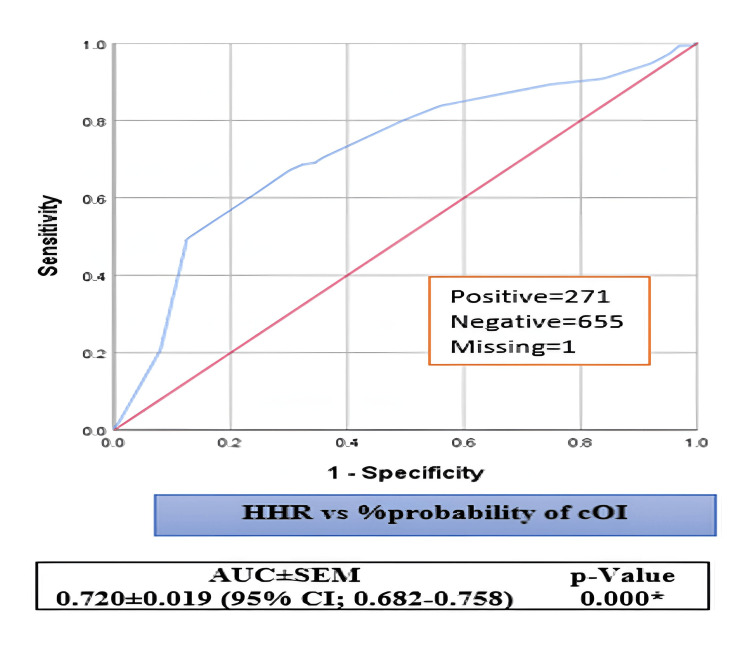
Receiver operating characteristic (ROC) test for HHR vs. probability of cOI positivity HHR: Hematocrit-to-hemoglobin ratio; ROC: receiver operating characteristic; cOI: composite outcome of interest; AUROCs: area under the ROC curves; SEM: standard error of mean; *: statistically significant The ROC test examined the AUROCs for HHR against the cOI. Higher %Prob of cOI values indicate stronger positive evidence for our tested prognosticators. The lower values of the independent variables suggest a more negative state (higher %Prob of naïve cOI)

On the other hand, our models account for the variability of the dependent variable to varying degrees, ranging from 22.5% to 32.1% and 10.7% to 15.3%, depending on whether we consult the Cox & Snell R^2^ or Nagelkerke R^2^ methods (Table 1). The area under the curves for the Na levels and HHR that were being studied were 0.789 0.018 (95% CI: 0.753-0.825) and 0.720 0.019 (95% CI: 0.682-0.758), compared to the chance of cOI positivity (Figures 1-2). In addition to the ROC illustrations, the positivity, negativity, and missing analyzed values for each of the aforementioned prognosticators were 271, 655, and 1, respectively.

**Table 1:** The binary logistic regression analysis for Jordanian intermittent hemodialysis patients from 2015 to 2022 reveals significant correlations between sodium level and hematocrit-to-hemoglobin ratio BLgR: Binary logistic regression; Na: sodium; HHR: hematocrit-to-hemoglobin ratio; KHMC: King Hussein Medical Center; SEM: standard error of mean; HDU: hemodialysis unit; RMS: Royal Medical Services; cOI: composite outcomes of interest The BLgR analysis was conducted for the investigated Na levels and the HHR against the cOI for Jordanian hemodialysis patients, to explore the degree of correlations, how much of the total variations in the dependent variable can be explained by the independent variables, and the quality of the prediction of the dependent variable. In this study, the higher %Prob of cOI is considered as a positive state, and the lower %Prob of cOI is considered as a negative state. For our tested prognosticator, the higher values indicate stronger evidence for the positive state (Higher %Prob of cOI), while the lower values of the tested independent variable indicate stronger evidence for the negative state (Higher %Prob of naïve cOI). The cOI include at least one of the predefined negatives occurring outcomes, of particular acute myocardial infarction, stroke, refractory hypertension, thrombosis of the dialysis graft, and all-cause mortality

Tested predictors	B ± SEM	Wald	Sig.	Exp (B)	95% C.I. for EXP(B)		χ^2^ (df) Sig	VR	%Cases
					Lower	Upper			
%Prob of cOI	e (109.36 - 0.849 × Na)/[1 + e (109.36 - 0.849 × Na)]								
Na (mEq/l)	-0.849 ± 0.064	175.158	0.000	0.428	0.377	0.485	(8) 42.344 0.000*	22.5%-32.1%	82.2%
Constant	109.36 ± 8.318	172.840	0.000	3.1 × 10^47^					
%Prob of cOI	e (16.033 - 6.388 × HHR)/[1 + e (16.033 - 6.388 × HHR)]								
HHR	-6.388 ± 0.655	95.143	0.000	0.002	0.000	0.006	(8) 63.329 0.000*	10.7%-15.3%	70.8%
Constant	16.033 ± 1.723	86.549	0.000	9.18 × 10^4^					

**Figure 3:**
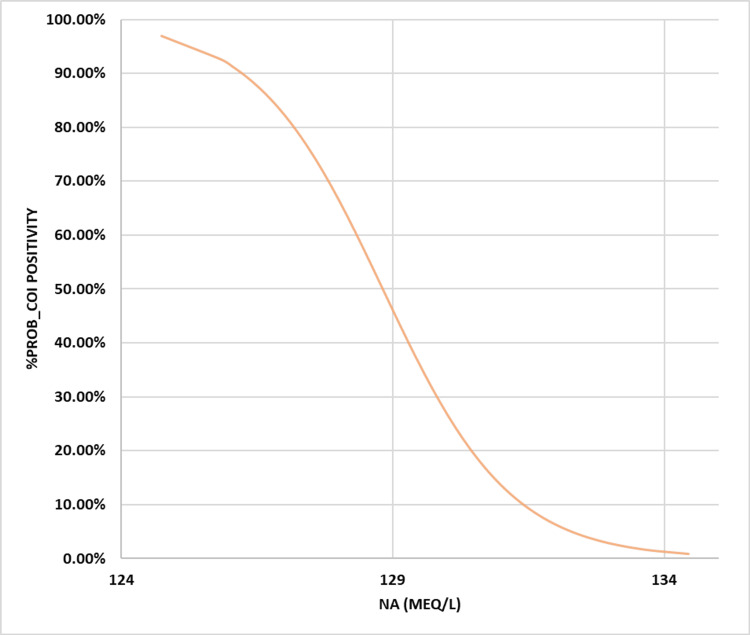
Binary logistic regression illustration for Na vs. probability for cOI positivity Na: Sodium; cOI: composite outcomes of interest; BLgR: binary logistic regression; KHMC: King Hussein Medical Center; HDU: hemodialysis unit; RMS: Royal Medical Services The BLgR analyses contrasted the higher probability of cOI (positive state and assigned as 1) with the lower probability (negative state and assigned as 0 for Jordanian patients who received intermittent hemodialysis at the KHMC HDU on the RMS, Amman, Jordan, between 2015 and 2022

**Figure 4:**
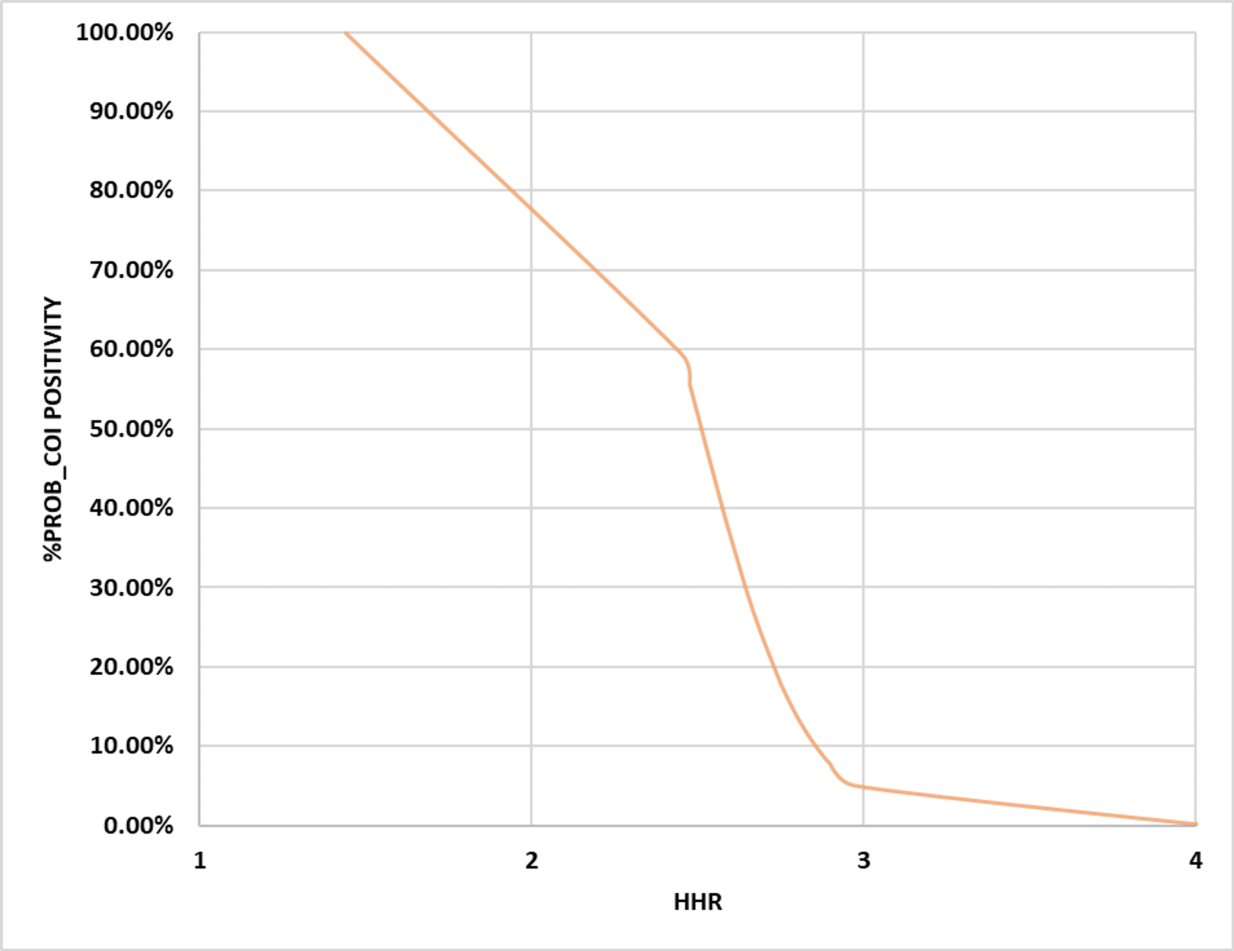
Binary logistic regression analysis for HHR vs. probability for cOI positivity BLgR: Binary logistic regression; HHR: hematocrit-to-hemoglobin ratio; KHMC: King Hussein Medical Center; HDU: hemodialysis unit; RMS: Royal Medical Services; cOI: composite outcomes of interest The BLgR analyses contrasted the higher probability of cOI (positive state and assigned as 1) with the lower probability (negative state and assigned as 0 for Jordanian patients who received intermittent hemodialysis at the KHMC HDU on the RMS, Amman, Jordan, between 2015 and 2022

In terms of the likelihood of finding inferior cOI instead of superior cOI, the best levels for each of the studied indices (Na and HHR) were 129.51 mEq/l and 2.555:1, respectively. Other sensitivity indices, like specificity, positive/negative predictive values, and accuracy index, showed different results than the ones listed above (67.2%, 69.92%, 48.02%, 83.73%, and 69.11%) (Table 2). These results were 66.1%, 87.18%, 68.06%, 86.12%, and 80.99%. The BLgR analysis revealed that any of the cOI had a 35.57% chance of occurring at the best Na concentration of 129.51 mEq/l and a 42.8% chance of happening at the best HHR of 2.555:1, as shown in Figures 3-4.

**Table 2:** The optimal cutoff points and other sensitivity indices for the investigated Na and HHR against the assessed cOI for Jordanian intermittent hemodialysis patients at the KHMC HDU on the RMS, Amman, Jordan, between 2015 and 2022 TPR: True positive rate or sensitivity; FPR: false positive rate; YI: Youden’s index; TNR: true negative rate or specificity; cOI: composite outcomes of interest; Na: sodium; HHR: hematocrit-to-hemoglobin ratio; KHMC: King Hussein Medical Center; HDU: hemodialysis unit; RMS: Royal Medical Services; PPV: positive predictive value; NPV: negative predictive value; NLR: negative likelihood ratio; PLR: positive likelihood ratio; AI: accuracy index The sensitivity analyses were processed on a total of 926 processed hemodialysis cases for the two investigated prognosticators against the higher probability of the cOI (positive state and assigned as 1) versus the lower probability of cOI (negative state and assigned as 0) to explore the optimal cutoff points and the other sensitivity analyses’ indices. The cOI include at least one of the predefined negatives occurring outcomes, in particular acute myocardial infarction, stroke, refractory hypertension, thrombosis of the dialysis graft, and all-cause mortality. In this study, the higher %Prob of cOI is considered as a positive state, and the lower %Prob of cOI is considered as a negative state. For our tested prognosticator, the higher values indicate stronger evidence for the positive state (higher %Prob of cOI), while the lower values of the tested independent variable indicate stronger evidence for the negative state (higher %Prob of naïve cOI)

Prognostic indicator	Cutoff	TPR	FPR	YI	TNR	PPV	NPV	NLR	PLR	AI
Na (mEq/l)	129.51	66.1%	12.8%	53.23%	87.18%	68.06%	86.12%	38.94%	515.05%	80.99%
HHR	2.555	67.2%	30.1%	37.08%	69.92%	48.02%	83.73%	46.97%	223.29%	69.11%

We added the LOD to the MLgR analysis and treated Na and HHR as categorical independent variables based on their optimal thresholds, as we did in the previous study. All of the examined independent variables became significant, except for HHR (p-value = 0.908). Furthermore, the model that is based on logistics yielded the most significant results, displaying a wider range of variability (30.4%-43.4%, ꭓ2(8)= 29.712). During this investigation, a higher percentage of cOI indicates a positive state, while a lower percentage indicates a negative state. Along with the higher LOD, the lower values of Na and HHR strengthen the case for the positive state (lower cOI) for the variables that are the focus of the study.

**Figure 5:**
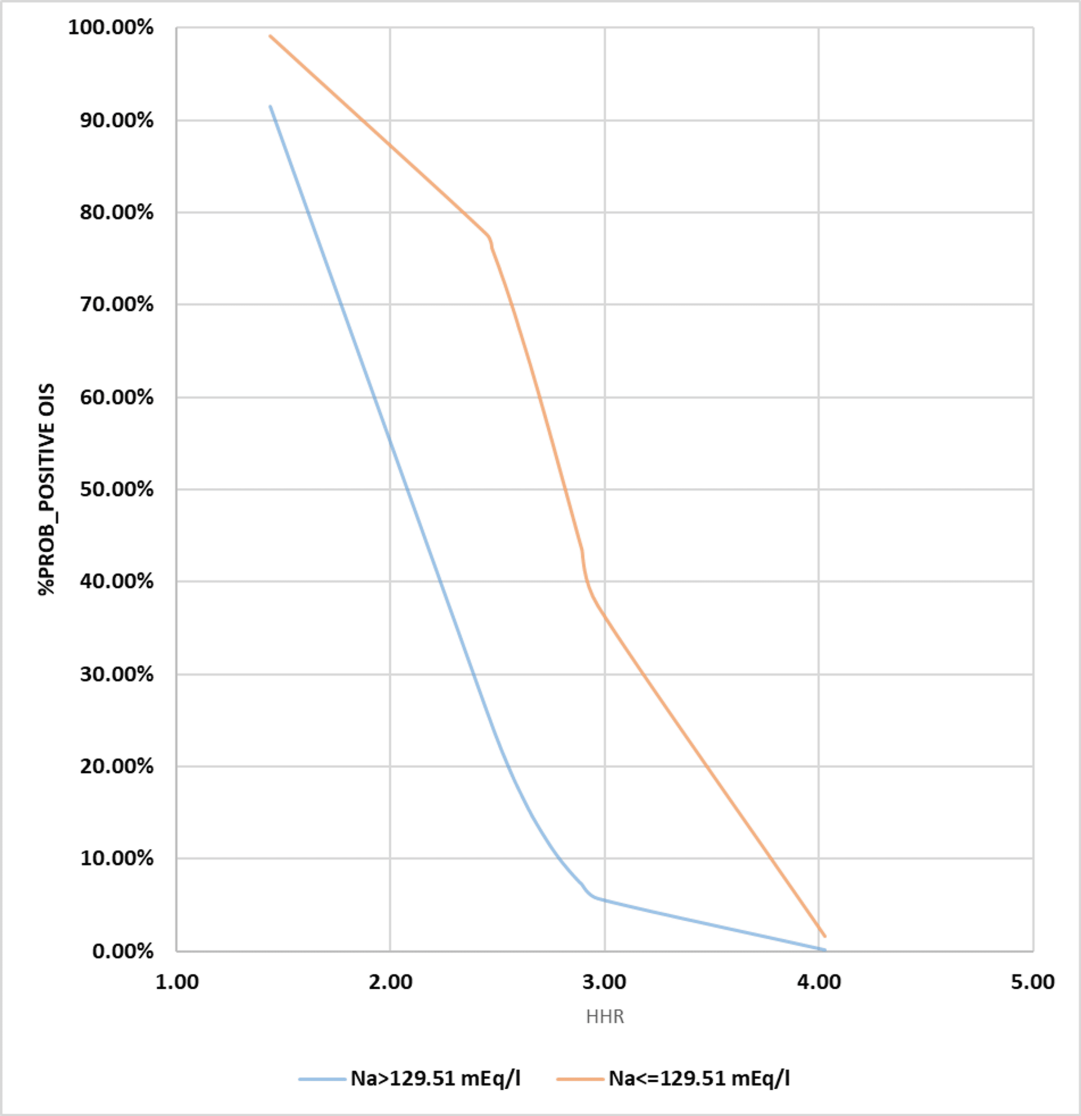
Multiple logistic regression illustration for both sodium states against probability for cOI positivity MLgR: Multiple logistic regression; cOI: composite outcomes of interest; Na: sodium; KHMC: King Hussein Medical Center; HDU: hemodialysis unit; RMS: Royal Medical Services Our constructed MLgR analyses’ illustration for the hemodialysis patients’ Na level against the probabilities of occurring at least one of the predefind cOI (acute myocardial infarction, stroke, refractory hypertension, thrombosis of the dialysis graft, and all-cause mortality) in the Jordanian hemodialysis patient who conducted their intermittent hemodialysis, between 2015 and 2022, at the HDU of the KHMC on the RMS, Amman, Jordan

**Figure 6:**
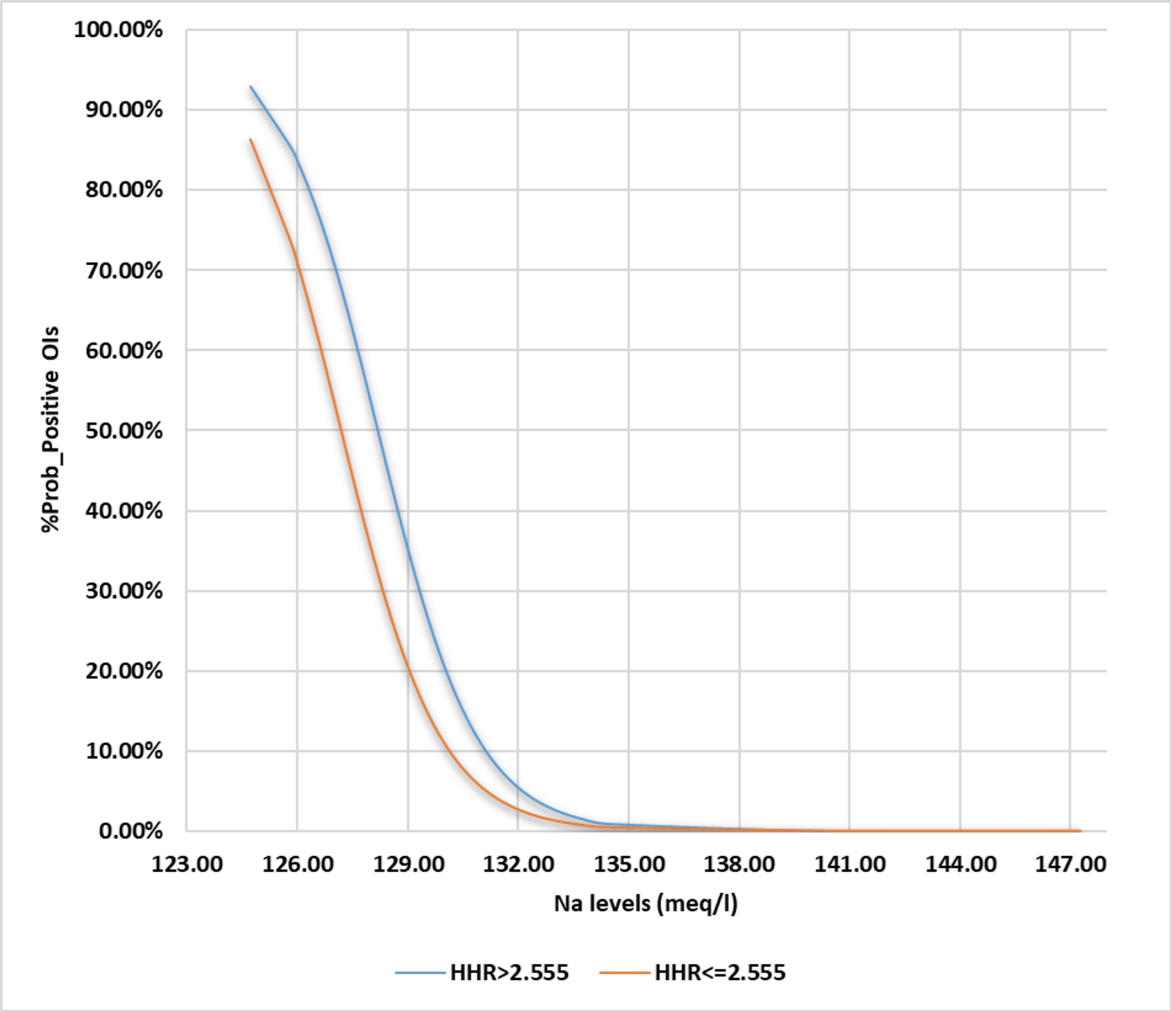
Multiple logistic regression illustration for both HHR states against probability for cOI positivity MLgR: Multiple logistic regression; cOI: composite outcomes of interest; KHMC: King Hussein Medical Center; HDU: hemodialysis unit; RMS: Royal Medical Services; HHR: hematocrit-to-hemoglobin ratio Our constructed MLgR analyses’ illustration for the hemodialysis patients’ HHR  against the probabilities of occurring at least one of the predefined cOI (acute myocardial infarction, stroke, refractory hypertension, thrombosis of the dialysis graft, and all-cause mortality) in the Jordanian hemodialysis patient who conducted their intermittent hemodialysis, between 2015 and 2022, at the HDU of the KHMC on the RMS, Amman, Jordan

**Figure 7:**
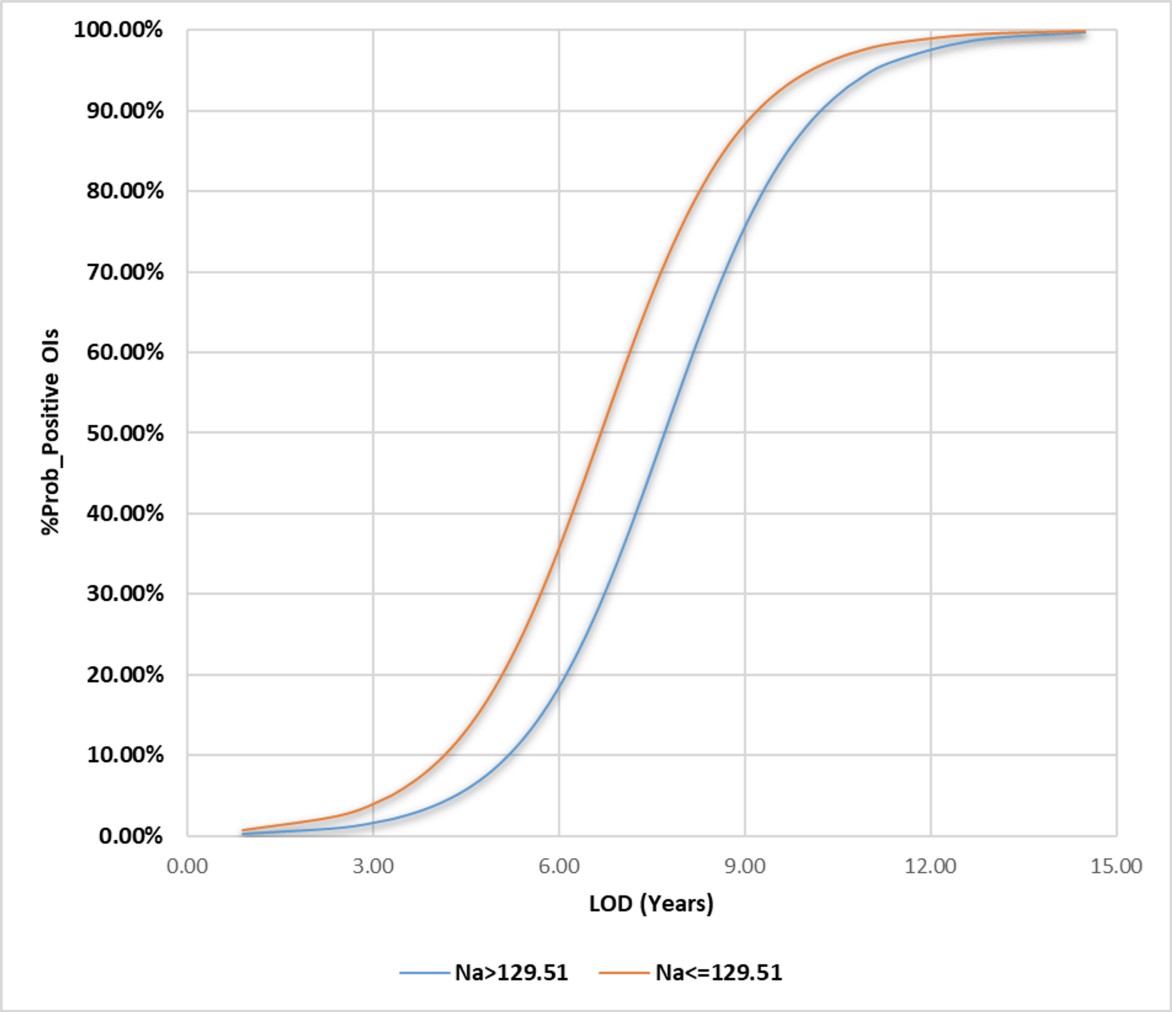
Multiple logistic regression illustration for the length of hemodialysis days against the probability for cOI positivity MLgR: Multiple logistic regression; cOI: composite outcomes of interest; KHMC: King Hussein Medical Center; HDU: hemodialysis unit; RMS: Royal Medical Services; LOD: length of hemodialysis Our constructed MLgR analyses’ illustration for the hemodialysis patients’ LOD, against the probabilities of occurring at least one of the predefined cOI (acute myocardial infarction, stroke, refractory hypertension, thrombosis of the dialysis graft, and all-cause mortality) in the Jordanian hemodialysis patient who conducted their intermittent hemodialysis, between 2015 and 2022, at the HDU of the KHMC on the RMS, Amman, Jordan

Stronger evidence favors the negative state with a lower LOD and higher values of the categorical independent variable under test, corresponding to a better coefficient of determination (cOI). We constructed these regressional-based models using the following expressions: [e (7.248 + 2.284 Na - 3.383 HHR)/[1 + e (7.248 + 2.284 Na - 3.383 HHR)]; [e (95.89 - 0.748 Na + 0.749 HR)/[1 + e (95.89 - 0.748 Na + 0.749 HR)]; and [e (-6.716 + 0.894 Na + 0.871 LOD)/[1 + e (-6.716 + 0.894 Na + 0.871 LOD)]. The constructed and illustrated models are expressed in Table 3 and Figures 5-7, respectively. Depending on using the Cox & Snell R^2^ or Nagelkerke R^2 ^methods, we successfully identified and explained the ranges of variation in the dependent variable. These ranges were as follows: 25.7%-36.7%, 24%-34.2%, and 30.4%-43.4%).

**Table 3:** The MLgR analyses for the patients’ HHR and Na, against the assessed cOI for the Jordanian hemodialysis patients who conducted their intermittent hemodialysis, between 2015 and 2022, at the HDU of the KHMC on the RMS, Amman, Jordan. SEM: standard error of mean; MLgR: multiple logistic regression; cOI: composiite outcomes of interest; Na: sodium; HHR: hematocrit-to-hemoglobin ratio; KHMC: King Hussein Medical Center; HDU: hemodialysis unit; RMS: Royal Medical Services; LOD: length of hemodialysis The MLgR analyses for the hemodialysis patients’ Na levels and the HHRs, were conducted in two vice versa situations against the probabilities of occurring at least one of the predefined cOI (acute myocardial infarction, stroke, refractory hypertension, thrombosis of the dialysis graft, and all-cause mortality) to explore the degree of correlations, how much of the total variations in the dependent variable can be explained by the independent variables, and the quality of the prediction of the dependent variable. Firstly, the HHR was processed as continuous independent variable, and the Na was processed as a categorical independent variable according to its previously explored optimal thresholds (>129.51 mEq/l=0, ≤129.51 mEq/l=1). In the alternative formulated situation, the HHR was processed as a categorical independent variable based on its optimal cutoff points (>2.555=0, ≤2.555=1), and the Na was processed as a continuous independent variable. In this study, the higher %Prob of cOI is considered as a positive state, and the lower %Prob of cOI is considered as a negative state. For our tested independent variables, the lower values of either Na and HHR and higher LOD indicate stronger evidences for the positive state (poorer cOI). However, the higher values of the tested categorical-based independent variable and the lower LOD indicate stronger evidences for the negative state (better cOI)

Tested predictors	B ± SEM	Wald	Sig.	Exp (B)	95% C.I. for EXP(B)		χ^2^ (df)	VR	%Cases
					Lower	Upper			
%Prob of cOI	=e (7.248 + 2.284 × Na - 3.383 × HHR)/[1+ e (7.248 + 2.284 × Na - 3.383 × HHR)]								
HHR	-3.383 ± 0.759	19.862	0.000	0.034	0.008	0.150	(7) 109.561 0.000*	25.7%-36.7%	81.0%
Na (0 or 1)	2.284 ± 0.183	155.672	0.000	9.812	6.854	14.046			
Constant	7.248	2.027	12.79	0.000					
%Prob of cOI	=e (95.89 - 0.748 × Na + 0.749 × HHR)/[1 + e (95.89 - 0.748 × Na + 0.749 × HHR)]								
HHR (0 or 1)	0.749 ± 0.178	17.712	0.000	2.114	1.492	2.995	(8) 62.763 0.000*	24%-34.2%	84.1%
Na (mEq/l)	-0.748 ± 0.068	120.375	0.000	.473	0.414	0.541			
Constant	95.89 ± 8.871	116.848	0.000	4.4 × 10^41^					
%Prob of cOI	=e (-6.716 + 0.894 × Na + 0.871 × LOD)/ [1 + e (-6.716 + 0.894 × Na + 0.871 × LOD)]								
HHR (0 or 1)	-0.097 ± 0.236	0.169	0.681	0.908	0.571	1.441	(8) 39.712 0.000*	30.4%-43.4%	83.9%
Na (0 or 1)	0.894 ± 0.271	10.843	0.001	2.445	1.436	4.162			
LOD (Years)	0.871 ± 0.133	43.145	0.000	2.390	1.843	3.099			
Constant	-6.716 ± 0.720	86.908	0.000	0.001					

## Discussion

Patients with severe hyponatremia on dialysis have a higher risk of death and heart disease. This is especially true if they have high potassium and low osmolarity. Clinical guidelines prioritize renal failure and electrolyte imbalance management. With severe hyponatremia, cardiovascular complications, and hemolysis, patient mortality increases. Acute or chronic renal failure complicates clinical management and lacks reliable outcome data for patients with severe electrolyte disturbances who require renal replacement therapy. Experts recommend various treatments for these conditions. The European Society of Intensive Care Medicine, European Society of Endocrinology, and European Renal Association-European Dialysis and Transplant Association have clinical practice guidelines for diagnosing and treating hyponatremia, which is common and has many treatments. These guidelines aid clinicians and prioritize patient outcomes [16].

A systematic review and meta-analysis of 1,116 studies from 2011 to 2020 found a significant association between hyponatremia and baseline and time-varying all-cause mortality in hemolysis patients. Hyponatremia independently predicted dialysis patients' all-cause and cardiovascular mortality, likely predicting complications. The study examined hyponatremia and worse outcomes in maintenance hemolysis patients undergoing ≥60 days. The study targeted patients undergoing ≥60 days of hemolysis. The study of 424 HD patients linked lower blood Na and potassium levels before HD to lower albumin, normalized protein catabolic rate (nPCR), and other illnesses. Doctors should be aware that hyponatremia increases all-cause mortality in maintenance hemolysis patients. Blood gas analyzers and core laboratory autoanalyzers showed moderate correlations for Na, potassium, glucose, Hgb, and Hct, but no agreement limits were found [17-18].

In a seven-year observational study of 631 stable hemolysis patients, low serum Na levels independently predicted all-cause and cardiovascular mortality across all age groups. The study found that these patients had hyponatremia due to fluid overload, inflammation, diet, and predialysis of blood glucose. Low serum Na in stable hemolysis patients may indicate poor glucose control. Predialysis glucose reports serum Na. This suggests more research on stable, healthy hemolysis patients' prognostic variables. Even with unknown causes, late hemoconcentration improved survival in decompensated heart failure patients. Continuous decongestion is necessary during treatment. The study included 422 patients with hemoconcentration heart failure. Shorter hospital stays, weight loss, the transition to oral diuretics, and higher loop doses were all associated with late hemoconcentration. Only hemoconcentration after late hospitalization improved decompensated heart failure survival, emphasizing the importance of sustained decongestion [19-20].

Preoperative serum Na concentrations above and below 142 and 138 mEq/L increased morbidity and mortality, indicating that this linear relationship should be rethought. This study found that hypernatremia (143-145 mEq/L) and borderline hyponatremia (135-137) affected perioperative mortality. Even within "normal" ranges, preoperative serum Na concentrations below 138 and above 142 mEq/L increased morbidity and mortality. Continuous Hct monitoring reduced intradialytic symptoms in 280 renal patients without changing treatment volumes or times. HD treats kidney disease, especially in low-income countries. A March 2019-February 2023 study of 280 patients revealed the need for HD among renal disease patients, particularly in low-income countries. Without changing treatment schedules or volume, continuous Hct monitoring reduced intradialytic symptoms by twice as much in hypotension-prone patients [21-22].

Hydrolysis significantly reduced serum osmolality, making early diagnosis and treatment crucial to survival. Lower Na levels increased interdialytic weight gain across ages and caused dialysis weight gain. The study examined hyponatremia causes in stable hemolysis patients of all ages. The study found no correlation between blood Na and Table 1 features. Low-Na groups had more diabetes nephropathy. In older patients, there was an inverse relationship between blood salt levels and weight gain between dialysis sessions. Low-blood-salt levels can predict prognosis in stable hemolysis patients of all ages. We do not know the cause of hyponatremia, but we should test stable HD patients with low blood salt for diabetes. There were differences between the Na quartiles in terms of body weight, diabetes, systolic blood pressure, interdialytic weight gain, total ultrafiltration, serum glucose, albumin, and creatinine, vascular access, and hemolysis type. The lowest serum Na quartile, Q1, had a significantly lower lean tissue index. Patients with sNa<136 mEq/l had a higher independent mortality risk (OR = 1.62) [23-24].

The KHMC at RMS in Amman, Jordan conducted the study on 1306 patients who participated in the HDU between 2015 and 2022. Among the 438 patients with end-stage renal disease (ESRD) who started or continued HD after 2018, 47.3% did not have any of the following health events during their years on dialysis: acute myocardial infarction, stroke, resistant hypertension, dialysis graft thrombosis, or death from any cause (with a significantly improved composite outcome index). The majority of patients with HD experienced HD for a period of four to seven years, with 708 individuals (76.5%) and 195 individuals (21.1%), respectively.

In our study, we employed BLgR analysis to forecast the probability of encountering inferior COI rather than superior COI. The ideal thresholds for Na levels and HHR were determined to be 129.51 mEq/l and 2.555:1, respectively. The ideal thresholds for these indices were 66.1%, 87.18%, 68.06%, 86.12%, and 80.99%. Furthermore, our findings demonstrated that reduced levels of Na and HHR, along with an elevated LOD, offer more compelling support for the higher cOI.

This study has limitations because retrospective descriptive studies cannot establish individual and composite causation between Na and hemoquotient values, as well as the probability of cOI. The inclusion of ESRD patients in the study may introduce selection bias and limited generalizability. Unlike other observational retrospective studies that didn't look at these factors, this one did. It looked at the complex relationship between Na levels and HHRs and the bad effects on patients.

## Conclusions

Our study concluded that Na level below 129.51 mEq/l, especially when the LOD was extended, is significantly associated with a higher probability of occurring at least one cOI. This study revealed that the probability was approximately 35.57% at the Na explored threshold. This study also showed that a drop in HHR below 2.555 may lead to a higher risk of at least one cOI, but these significant negative effects were negligible when considering the LOD. According to this study, which was conducted on Jordanian HD patients, we expect to monitor Na levels above 129.51 mq/l, especially when the patients' LOD is prolonged.
